# Impact of DST (Daylight Saving Time) on Major Trauma: A European Cohort Study

**DOI:** 10.3390/ijerph182413322

**Published:** 2021-12-17

**Authors:** André Nohl, Christine Seelmann, Robert Roenick, Tobias Ohmann, Rolf Lefering, Bastian Brune, Veronika Weichert, Marcel Dudda, The TraumaRegister DGU

**Affiliations:** 1 Department of Emergency Medicine, BG Klinikum Duisburg, 47249 Duisburg, Germany; 2 Emergency Medical Services, Fire Brigade Oberhausen, 46047 Oberhausen, Germany; 3 Department of Trauma, Hand and Reconstructive Surgery, University Hospital Essen, 45147 Essen, Germany; bastian.brune@uk-essen.de (B.B.); marcel.dudda@uk-essen.de (M.D.); 4 Helicopter Emergency Medical Service (HEMS), 47249 Duisburg, Germany; veronika.weichert@bg-klinikum-duisburg.de; 5 Research Department, BG Klinikum Duisburg, 47249 Duisburg, Germany; christine.seelmann@bg-klinikum-duisburg.de (C.S.); tobias.ohmann@bg-klinikum-duisburg.de (T.O.); 6 Clinic for Arthroscopic Surgery, Sports Traumatology and Sports Medicine, BG Klinikum, 47249 Duisburg, Germany; robert.roenick@bg-klinikum-duisburg.de; 7 Institute for Research in Operative Medicine (IFOM), University of Witten/Herdecke, 51109 Cologne, Germany; rolf.lefering@uni-wh.de; 8 Emergency Medical Services, Fire Brigade Essen, 45139 Essen, Germany; 9 Department of Trauma Surgery, BG Klinikum Duisburg, 47249 Duisburg, Germany; 10 Committee on Emergency Medicine, Intensive Care and Trauma Management (Sektion NIS) of the German Trauma Society (DGU), 10623 Berlin, Germany

**Keywords:** DST, daylight saving time, major trauma

## Abstract

(1) Background: Approximately 73 countries worldwide implemented a daylight saving time (DST) policy: setting their clocks forward in spring and back in fall. The main purpose of this practice is to save electricity. The aim of the present study was to find out how DST affects the incidence and impact of seriously injured patients. (2) Methods: In a retrospective, multi-center study, we used the data recorded in the TraumaRegister DGU^®^ (TR-DGU) between 2003 and 2017 from Germany, Switzerland, and Austria. We compared the included cases 1 week before and after DST. (3) Results: After DST from standard time to summertime, we found an increased incidence of accidents of motorcyclists up to 51.58%. The result is consistent with other studies. (4) Conclusion: However, our results should be interpreted as a tendency. Other influencing factors, such as time of day and weather conditions, were not considered.

## 1. Introduction

In 2021, 73 nations worldwide conducted DST transitions in a biannual manner, adjusting the clock in order to establish a scenario in which the daylight is maximal utilized for waking activity. 

In 2019, the EU Parliament accepted the EU Commission’s proposal to abolish the time change in 2021. However, nothing has happened since then. The changeover is now to remain until at least 2026. The basis for the abolition of the clock changeover was a survey of people living in the EU.

In this (non-representative) online survey, 84 percent voted in favor of ending the switch between summer and winter time. A total of 4.6 million people took part, two-thirds of them from Germany [1].

The first transition takes place in spring, when the clock is set one hour forwards, and the second in autumn, when the clock is set one hour back again. The main arguments for this adjustment were economic reasons (energy saving) and changing illumination conditions for peak traffic density to likely avoid accidents due to bad light conditions in the evening, when the number of traffic accidents are elevated due to driver fatigue. The rationale of this approach is to shift one extra hour of daylight to the evening hours to compensate for the driver’s lack in concentration.

There is evidence that especially the DST transition might have a negative impact on people’s health, leading to sleep deprivation and circadian misalignment [2,3,4,5,6,7]. A study by Kantermann et al. suggests that the human circadian system does not adapt to daylight saving time and that its seasonal adaptation to changing photoperiods is disrupted by the introduction of daylight saving time. This disruption could also affect other aspects of human seasonal biology [3]. Jin et al. used an empirical approach to exploit the end of daylight saving time in a quasi-experimental setting on a daily basis. Due to the time reset in the fall, sleep time was extended by one hour. The study group found significant health benefits as hospital admissions decreased. For example, hospital admissions for cardiovascular disease decreased by 10 per day per million population. Using an event study approach, they found that the effect continued for four days after the time change. Admissions for heart attacks and injuries also showed the same characteristic four-day decline [8]. Toro et al. analyzed the effects of acute light sleep deprivation and circadian rhythm disturbances due to daylight saving time on the incidence of acute myocardial infarction using daily data for Brazil. They found robust evidence of a significant increase (7.4–8.5%) in the number of acute myocardial infarctions in Brazilian states with a time change to summer time but no statistical relationship between states without a time change [9].

A possible connection between DST transition and fatal traffic accidents is controversially discussed in literature. Fritz et al. (2020), for example, found acute consequences of the DST transition in spring on traffic accidents in a chronobiologic context [10]. Here, the spring DST transition increased fatal motor vehicle accidents by 6% in the DST week, whereas the fallback transition in autumn to Standard Time had no effects. Lahti et al. 2011 found that the sleep deprivation after DST transition is not harmful enough to have an influence on the incidence of occupational accident rates [11]. In a recent systematic review, Carey and Sarma addressed the topic about the impact of DST on road traffic collision risk [12]. A total of 24 studies were included in this overview. The complex picture emerging from this review showed day- and time-dependent potentially positive or negative short-term effects of DST and a possible positive long-term effect. 

Because of inconsistent findings and conclusions across the mostly heterogeneous studies, no conclusion about a positive or negative overall impact of DST on traffic accidents could be drawn. 

Traffic accidents represent only a part of all trauma patients. As a whole, trauma accounts for 10% of deaths worldwide and is the leading cause of death in people younger than 40 years [13]. Therefore, advancements in the prevention of accidents are inevitable. However, external factors such as time of the day, day of the week, and seasons are thought to affect trauma events [14]. Here, it seems obvious that the incidence of traumata might be affected by DST transition markedly. 

Previous studies concentrated on the analysis of the impact of DST on traffic accidents, but none investigated the incidence of trauma that occurred from other reasons. 

In this present study, our approach was to analyze the very extensive database of the TraumaRegisters DGU^®^, which has not been used for this purpose thus far and provides an excellent data collection to address the question if DST has an impact on the incidence and impact on major trauma. 

## 2. Materials and Methods

### 2.1. Data Set

The TraumaRegister DGU^®^ (TR-DGU, AUC - Akademie der Unfallchirurgie GmbH, Munich, Germany) of the German Trauma Society was founded in 1993 to create a multicenter database for pseudonymized and standardized documentation of severely injured patients for quality assurance and research [15]. 

Participating hospitals are predominantly located in Germany (90%), but an increasing number of hospitals from other countries have also started to contribute their data (e.g., Austria, Belgium, Finland). Currently, approximately 30,000 cases from more than 650 hospitals are entered into the database annually. Participation in TR-DGU is voluntary; however, hospitals associated with TraumaNetzwerk DGU^®^ are required to enter at least a basic dataset for reasons of quality assurance.

Documentation in the TR-DGU includes detailed information on:

- Demographics;
- Injury patterns;
- Comorbidities;
- Prehospital data;
- Course of hospital treatment, including intensive care unit, relevant laboratory findings, transfusions, and interventions;
- Outcome.

Inclusion criteria for the TR-DGU are admission to hospital via the emergency department followed by intensive care or admission to hospital with vital signs and death before admission to the intensive care unit (ICU).

**Figure 1:**
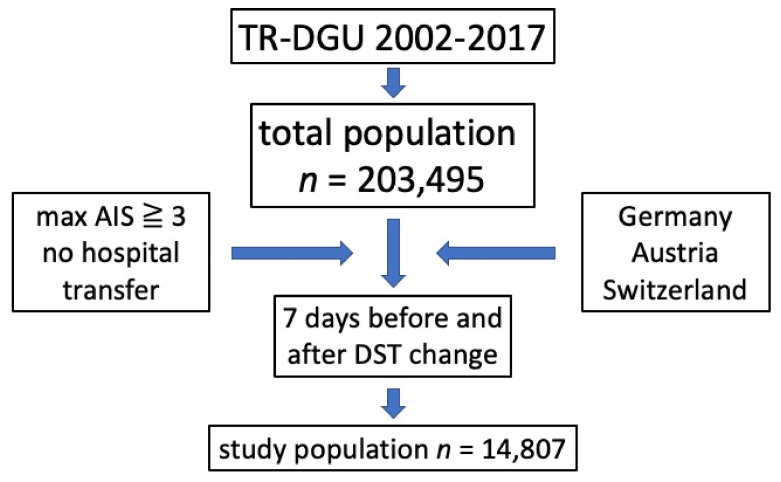
Inclusion criteria; max = maximum; AIS = abbreviated injury score; DST daylight saving time.

The infrastructure for documentation and data management is provided by the AUC - Academy of Trauma Surgery, a society affiliated with the German Trauma Society. Scientific management is provided by the Committee on Emergency Medicine, Intensive Care and Trauma Management (Sektion NIS) of the German Trauma Society. The scientific evaluation of the data is performed according to a peer-review process defined in the publication guideline of the TR-DGU [15]. The present study complies with the publication guideline of the TR-DGU and is registered under the TR-DGU project ID 2018-047. The inclusion criteria of our study are shown in Figure 1. 

Permission by the ethics committee (Ärztekammer Nordrhein/Medical Association North Rhine; no. 310/2018).

### 2.2. Statistics

The day of DST change was excluded (usually a Sunday), and a time period of 7 days before and after DST change in spring and autumn was selected for comparison (the comparison of 7 days was chosen because the direct comparison of individual days showed a low number of cases; in the studies cited, the procedure was comparable). Both pre- and post-change phases thus contained each weekday once. Data are presented as number of cases with percentage for counts, and as mean with standard deviation (SD) for metric data. In seriously skewed data, median and inter-quartile range were given instead. Observed differences were evaluated with the chi-squared test or the Mann–Whitney *U*-test. 

Statistical analysis was performed using SPSS Statistical software (Version 27.0, IBM Inc., Armonk, NY, USA). The level of statistical significance was set at *p* < 0.05.

The RISC II score was developed and validated using TR-DGU data and represents a summary of the 13 variables, including pattern and severity of injuries, age, sex, prior diseases, and initial physiology [16].

## 3. Results

A total of 14,807 trauma patients were included in the study. The mean age was 51 (±22) years. The majority were males (71%). In Table 1, the mechanism of accident 1 week before and 1 week after the time change are listed. More traffic accidents occurred after the time change (*n* = 3459 vs. 3582), but it was not statistically significant (*p* = 0.131). The mean ISS was higher after the time change. Although, this difference did not reach the significance level, we observed a strong tendency (*p* = 0.052). There was a noticeable increase in the number of motorcycle and bicycle accidents during the time change from spring to summer (motorcycle *n* = 349 vs. 529, increase of 51.58%; bicycle *n* = 245 vs. 280, increase of 14.29%). After the time change in autumn, these incidents decreased in number compared to the previous week.

Table 2 shows demographics and the distribution of different injury regions before and after the time change. Increased deaths after time change *n* (%) = 900 (12.2) vs. 950 (12.8). The age, injury severity, expected mortality (RISC II), ICU, and hospitalization days are shown in Table 3.

## 4. Discussion

The results of this study showed an increased incidence of traffic accidents (bicycle and motorcycle) after the spring DST. Motorcycle accidents showed an increase of 51.58%, whereas accidents involving motorcyclists slightly decreased again after the time change in autumn. However, it is worth mentioning that many motorcyclists have summer license plates from March to October. Central European Summer Time begins on the last Sunday in March at 2:00 CET. Therefore, there is an interval of over 4 weeks from the seasonal registration of the motorcycles to the change of time.

However, a possible cause could also be the general trend that motorcycle and bicycle accidents occur more frequently in the summertime and decrease again in the winter. External influences, such as time of day, lighting conditions, days of the week, weather conditions, and temperatures were not considered in our study. 

The number of registered motorcycles in Germany has risen steadily, reaching 4.31 million in 2017. We detected 10 of the busiest highways in the most populous state of North Rhine-Westphalia and compared 10 automatic counting stations for traffic measurement between March 2017 and April 2017. The 10 automatic counting stations counted 5848 motorcycles in March 2017 and 6362 motorcycles in April 2017. Thus, there is an increase of 8.79% in the number of motorcycles counted on the 10 busiest highways in North Rhine-Westphalia from March to April. The increase of 8.79% more motorcycles counted from March to April contrasts with the 51.58% increase in motorcyclists seriously injured after the time change in spring. Although only 10 measuring points were evaluated, the sample of a total of 12,210 was representative. However, a sample of a German state is compared here with a European cohort in our study [17].

A comparable study by Pape-Köhler et al. (2014) showed that the time of day shows a high variation in the incidence of trauma. In particular, the frequency of accidents increased during rush hour [14]. However, these external factors could also have an influence on accident frequency. In addition, our data do not show whether the motorcyclists caused the traffic accidents themselves or were harmed by other road users.

Importantly one should take into account that due to our inclusion criteria, only accidents with serious injuries were considered. Thus, no conclusion can be drawn about the general frequency of accidents.

Fritz et al. (2020) showed similar results in their study. The study group was able to show that the incidence of serious car accidents increased by 6% after the time change in spring. They attributed the result to sleep deprivation due to the one-hour time shift. Here, the frequency of accidents was increased, especially in the early morning hours. The research team concluded that leaving out the time change could prevent up to 27 serious traffic accidents annually [10]. 

Besides the disturbance of the circadian rhythm, another reason could be the fact that due to the time shift by one hour later, it is correspondingly darker in the early morning and thus an increased accident frequency could be explained.

In a study by Robb et al. (2018), an increased accident frequency was proven in the first 2 days after the time change in New Zealand [18].

Another study from Spain showed that the time change is associated with fatal accidents of 1.5 people per year in 52 Spanish capital cities [19].

What is slightly noticeable in our study is the increased accident frequency explicitly among motorcyclists. There are a number of confounding factors that influence the frequency of motorcycle accidents. The biggest disruptive factors are certainly the winter break and a colder season at the beginning of the motorcycle season. It has been proven that the frequency of accidents increases with age (young, inexperienced riders, as well as seniors), general riding experience and frequency, weight of the motorcycle, male gender, weather conditions, etc. [20].

However, here, the period of 1 week before and 1 week after the time change was quite short to conclude. 

Thus, according to our results, if the time change were eliminated, up to 12 serious motorcycle accidents could be prevented each year if these accidents were indeed related to DST. 

However, we cannot confirm this statement with a probability bordering on certainty. Nevertheless, due to the external influences not taken into account and the lack of statistical significance, it should rather be seen as a tendency. Our result is certainly not conclusive enough for a clear recommendation.

### Strengths and Limitations

Due to the high number of cases and the study period of almost two decades, our study has a high level of representativeness.

This study has several limitations. It is a retrospective analysis. 

Patients who died in the prehospital setting are not included in the TraumaRegister DGU^®^. Data from patients who were admitted to the hospital without activation of the trauma team are not captured by the TraumaRegister DGU^®^. Another limitation of this study is that treatment restrictions, for example due to a living will, were not recorded. The participation of hospitals in the TraumaRegister DGU^®^ is voluntary [15].

## 5. Conclusions

There is a noticeable increase in the number of motorcycle and bicycle accidents during the DST from standard to summer time. Our results should be understood as a tendency, since various influencing factors, such as times of day and weather, were not taken into account.

## Figures and Tables

**Table 1:** Impact of daylight saving time change in patients with major trauma.

Accident History, *n* = 14,488					
			Pre-Week	Post-Week	Total
DST standard to summer time		car/lorry, *n* (%)	758 (21.9)	778 (20.8)	1534 (21.3)
		motorcycle, *n* (%)	349 (10.1)	529 (14.1)	878 (12.2)
		bicycle, *n* (%)	245 (7.1)	280 (7.5)	525 (7.3)
		pedestrians, *n* (%)	218 (6.3)	209 (5.6)	427 (5.9)
		high fall > 3 m, *n* (%)	589 (17.0)	610 (16.3)	1199 (16.6)
		low fall < 3 m, *n* (%)	869 (25.1)	870 (23.2)	1739 (24.1)
		others, *n* (%)	430 (12.4)	472 (12.6)	902 (12.5)
		total, *n*	3456	3748	7204
DST summer to standard time		car/lorry, *n* (%)	863 (23.0)	830 (23.5)	1693 (23.2)
		motorcycle, *n* (%)	412 (11.0)	319 (9.0)	731 (10.0)
		bicycle, *n* (%)	288 (7.7)	244 (6.9)	532 (7.3)
		pedestrians, *n* (%)	243 (6.5)	303 (8.6)	546 (7.5)
		high fall > 3 m, *n* (%)	653 (17.4)	638 (18.1)	1291 (17.7)
		low fall < 3 m, *n* (%)	866 (23.1)	803 (22.8)	1669 (22.9)
		others, *n* (%)	430 (11.5)	392 (11.1)	822 (11.3)
		total, *n*	3755	3529	7284
Total		car/lorry, *n* (%)	1619 (22.5)	1608 (22.1)	3227 (22.3)
		motorcycle, *n* (%)	761 (10.6)	848 (11.7)	1609 (11.1)
		bicycle, *n* (%)	533 (7.4)	524 (7.2)	1057 (7.3)
		pedestrians, *n* (%)	461 (6.4)	512 (7.0)	973 (6.7)
		fall > 3 m, *n* (%)	1242 (17.2)	1248 (17.1)	2490 (17.2)
		fall < 3 m, *n* (%)	1735 (24.1)	1673 (23.1)	3408 (23.5)
		others, *n* (%)	860 (11.9)	864 (11.9)	1724 (11.9)
		total, *n* (%)	7211	7277	14,488

DST = daylight saving time.

**Table 2:** Cross tabulation.

Impact of DST in Major Trauma				
		**Pre-DST**	**Post-DST**	***p*-Value**
Traffic accident, *n* (%)	no	3752 (52.0)	3695 (50.8)	*p* = 0.131
	yes	3459 (48.0)	3582 (49.2)	
Blunt/penetrating trauma, *n* (%)	blunt	6749 (95.8)	6808 (95.6)	*p* = 0.595
	penetrating	295 (4.2)	311 (4.4)	
Sex, *n* (%)	female	2172 (29.6)	2066 (27.8)	*p* = 0.017
	male	5175 (70.4)	5367 (72.2)	
Age 70+ years, *n* (%)	<70	5459 (74.4)	5502 (74.2)	*p* = 0.778
	>70	1877 (25.6)	1912 (25.8)	
Died, *n* (%)	no	6482 (87.8)	6495 (87.2)	*p* = 0.325
	yes	900 (12.2)	950 (12.8)	
AIS head ≥ 3, *n* (%)	<3	3968 (58.9)	3993 (53.6)	*p* = 0.746
	≥3	3394 (46.1)	3452 (46.4)	
AIS thorax ≥ 3, *n* (%)	<3	3898 (52.9)	3942 (52.9)	*p* = 0.999
	≥3	3464 (47.1)	3503 (47.1)	
AIS abdomen ≥ 3, *n* (%)	<3	6384 (86.7)	6473 (86.9)	*p* = 0.681
	≥3	978 (13.3)	972 (13.1)	
AIS extremities ≥ 3, *n* (%)	<3	5166 (70.2)	5106 (68.6)	*p* = 0.036
	≥3	2196 (29.8)	2339 (31.4)	

AIS = abbreviated injury scale, *DST* = daylight saving time.

**Table 3:** Impact of DST in major trauma—spring and autumn.

	Pre-DST	Post-DST	*p*-Value
Age, mean (SD)	51 (22)	51 (22)	0.501
ISS, mean (SD)	21.8 (11.7)	22.3 (12.1)	0.052
Prognosis based on RISC II, mean %	13.2	13.4	0.439
ICU days, median (IQR)	2 (1–8)	3 (1–8)	0.035
Hospital days, median (IQR)	13 (5–23)	13 (6–23)	0.457

DST = daylight saving time, ICU = intensive care unit, ISS = Injury Severity Score, IQR = interquartile range, RISC II = Revised Injury Severity Classification II, SD = standard deviation.

## Data Availability

Data are available from andre.nohl@bg-klinikum-duisburg.de.

## Abbreviations

AIS: abbreviated injury scale
DST: daylight saving time
ICU: intensive care unit
ISS: Injury Severity Score
IQR: interquartile range
RISC II: Revised Injury Severity Classification II
